# Soluble antigens from the neurotropic pathogen *Angiostrongylus cantonensis* directly induce thymus atrophy in a mouse model

**DOI:** 10.18632/oncotarget.17836

**Published:** 2017-05-12

**Authors:** Zhen Liu, Dong-Ming Su, Zi-Long Yu, Feng Wu, Rui-Feng Liu, Shi-Qi Luo, Zhi-Yue Lv, Xin Zeng, Xi Sun, Zhong-Dao Wu

**Affiliations:** ^1^ Department of Parasitology of Zhongshan School of Medicine, Sun Yat-Sen University, Guangzhou, China; ^2^ Key Laboratory of Tropical Disease Control, Ministry of Education, Guangzhou, China; ^3^ Provincial Engineering Technology Research Center for Diseases-Vectors Control, Guangzhou, China; ^4^ Institute for Molecular Medicine, University of North Texas Health Science Center, Fort Worth, TX, USA

**Keywords:** Angiostrongylus cantonensis, central nervous system, thymic atrophy, soluble antigens, intrathymic injection, Gerotarget

## Abstract

The nematode *Angiostrongylus cantonensis* (*A.C*.) is a neurotropic pathogen; stage-*III* larva invade the human (non-permissive host) central nervous system (CNS) to cause eosinophilic meningitis or meningoencephalitis accompanied by immunosuppression. In an *A.C*.-infectedmouse (another non-permissive host) model, CNS damage-associated T cell immune deficiency and severe inflammation were proposed to result from activation of the hypothalamic-pituitary-adrenal (HPA) axis. However, glucocorticoids are anti-inflammatory agents. Additionally, while defects in thymic stromal/epithelial cells (TECs) are the major reason for thymic atrophy, TECs do not express the glucocorticoid receptor. Therefore, activation of the HPA axis cannot fully explain the thymic atrophy and inflammation. Using an *A.C*.-infected mouse model, we found that *A.C*.-infected mice developed severe thymic atrophy with dramatic impairments in thymocytes and TECs, particularly cortical TECs, which harbor CD4^+^CD8^+^ double-positive thymocytes. The impairments resulted from soluble antigens (sAgs) from *A.C*. in the thymuses of infected mice, as intrathymic injection of these sAgs into live mice and the addition of these sAgs to thymic cell culture resulted in thymic atrophy and cellular apoptosis, respectively. Therefore, in addition to an indirect effect on thymocytes through the HPA axis, our study reveals a novel mechanism by which *A.C*. infection in non-permissive hosts directly induces defects in both thymocytes and TECs via soluble antigens.

## INTRODUCTION

*Angiostrongylus cantonensis* (*A.C*.) is a zoonotic nematode. When *A.C.-*infected larvae (stage-*III* larva) invade suitable hosts, such as rats, they are transported via the blood to the brain and finally to the pulmonary arteries of the hosts, where they mature to adulthood and oviposit to complete their life cycles. However, when the larvae invade non-permissive hosts, such as humans and mice, the infective larvae cannot travel to the lung to finish their life cycles; instead, they remain in the central nervous system (CNS) until their death. In the non-permissive host CNS, the larvae cannot develop further and migrate, leading to severe inflammation in the brain. *A.C*.-induced acute inflammation in the brain is characterized by eosinophilic meningitis or meningoencephalitis [1]. Currently, angiostrongyliasis has been reported worldwide, particularly in tropical countries, and is regarded as a serious public health problem. In recent years, several outbreaks of human angiostrongyliasis have been reported in China and other countries, and the harmful effects on human health have been recognized [2–5]. Interestingly, *A.C*. larvae do not invade the thymus directly in these non-permissive hosts, although angiostrongyliasis can cause severe thymic atrophy. These changes are associated with immunosuppression/immunodeficiency and systemic inflammatory responses [6].

The thymus is a central T-lymphocyte organ of the immune system that generates functional naive T cells for cellular immunity and depletes self-reactive T cells for self-tolerance [7, 8]. The thymus is very sensitive to insults, such as whole-body viral (influenza) [9], parasitic (*Trypanosoma cruzi*) [10], and fungal infections (*Paracoccidioides brasiliensis*) [11], which are common agents of infectious diseases [12]. Typically, the thymus shows acute atrophy after insult, resulting in modifications of thymic structure and alterations in naive T cell output to the periphery. These changes promote immune suppression/secondary immunodeficiency and autoimmune/inflammatory responses due to reduced regulatory molecules, such as regulatory T cells (Tregs), which disturb the systemic immune response and inhibit the disposal of pathogens [13, 14]. A recent report showed that *A.C.* infection induced acute thymic atrophy via apoptosis-induced depletion of CD4^+^CD8^+^ (double positive, DP) thymocytes in non-permissive hosts [6]. CNS damage-associated activation of the hypothalamic-pituitary-adrenal (HPA) axis was proposed to play a major role in the disruption of thymocyte development, as thymocytes express the glucocorticoid receptor (GR). However, blockade of GRs using RU486 did not prevent thymocyte depletion during *A.C.* infection [6]. Additionally, defects in thymic stromal/epithelial cells (TECs) constitute the major reason for thymic atrophy, yet TECs, which form a three-dimensional network structure to support thymocyte development [15, 16], do not express GRs [17]. Furthermore, although glucocorticoids (GCs) stimulate pleiotropic changes and may cause side effects, they are steroidal anti-inflammatory agents with anti-inflammatory effects [18], while *A.C*. infection induces severe inflammation. Thus, although activation of the HPA axis has been proposed as a potential mechanism for thymic atrophy, it likely has an indirect effect. Whether *A.C.* infection can directly induce immune system (organ and cells) deficiency and whether there are other mechanisms involved in thymic atrophy, especially depletion of the CD4^+^CD8^+^ DP thymocytes induced by *A.C.* infection in non-permissive hosts, are questions that warrant further study.

In this study, we systematically and comprehensively demonstrated that *A.C.* infection induces strong thymic atrophy. This resulted in dramatic defects in thymocytes and TECs as well as disruption of the thymic structure. We found that infection-induced changes in the cellular and molecular characteristics of TEC subsets disrupted the thymic microenvironment and severely hampered the development of thymocytes, especially DP cells, by enhancing apoptosis. We also found that these changes in TECs were caused by *A.C*.-secreted soluble antigens (sAgs), which directly induced thymic damage. Therefore, in addition to revealing an indirect effect of the HPA axis, our study is the first to provide a direct mechanism by which *A.C.* infection in non-permissive hosts induces immune system deficiency via soluble antigens.

## RESULTS

### Severe thymic atrophy was observed in mice infected with A.C

After *A.C.* infection, obvious pathological changes in the brains of mice (non-permissive hosts) were observed by histological examination and compared with those of the control groups. Hemorrhages (black arrows) and inflammatory cell infiltration (green arrows) were significantly aggravated at 21 days post-infection (dpi) (Supplementary Figure S1). *A.C*. larvae were detected in the brain tissue [19], but not in the thymuses, of infected mice. These results from our experimental model confirmed previous findings that *A.C*. larvae directly invade the brains of hosts and that infection induces substantial inflammation in the brain.

**Figure 1:**
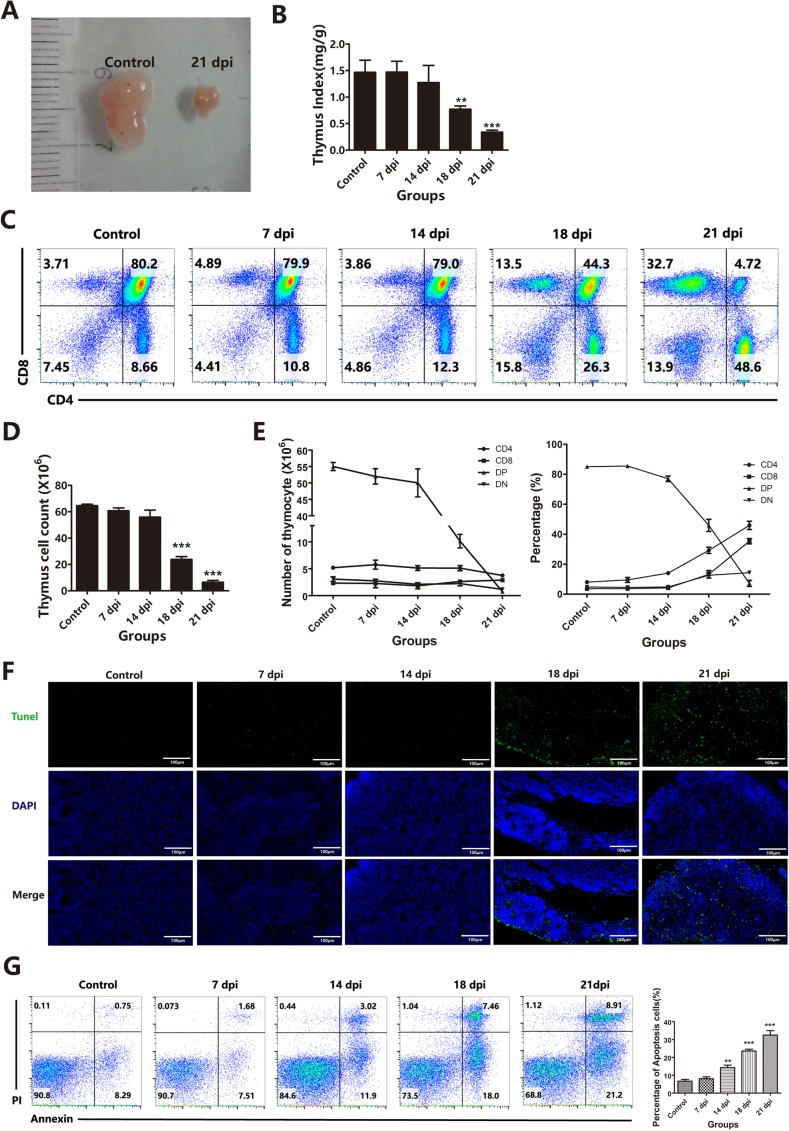
Thymic atrophy was associated with a dramatic decrease in DP thymocytes induced by *A. cantonensis* invasion into the brain **A**. Morphology of control and *A. cantonensis* (*A.C*.)-infected (21 dpi) mouse thymuses. **B**. Changes in the thymus index in control (non-infected) or 7, 14, 18 or 21 dpi thymuses (*n* = 5). **C**. The proportions of thymocyte subsets (DN, SP and DP) were analyzed using flow cytometry in control (non-infected) or 7, 14, 18 or 21dpi thymuses (*n* = 5). **D**. Changes in the number of total thymocytes in control (non-infected) or 7, 14, 18 or 21 dpi thymuses (*n* = 5). **E**. Changes in the number and percentage of DN, SP and DP thymocytes from control (non-infected) or 7, 14, 18 or 21 dpi thymuses (*n* = 5). **F**. In situ detection of apoptosis in thymuses using the In Situ Cell Death Detection Kit (TUNEL assay) (original magnification, x10). The green stain represents DNA fragmentation of apoptotic cells, and the blue stain shows the nuclei. **G**. The apoptotic thymocytes were identified by Annexin-V and PI staining. The percentages indicate the proportions of apoptotic cells. Data are representative of at least three independent experiments. The data are presented as the mean±S.D. **p < 0.01 and ***p < 0.001.

According to previous reports, *A.C*. infection can also induce thymic atrophy [6]. Strikingly, we also found significant thymic atrophy in our experimental model, which was observed in severely infected mice in our study. At 21 dpi, the thymus showed significant atrophy with a dramatic reduction in thymic mass (Figure 1A), and the thymus indices [weight of thymuses (mg)/body weight (g)] were significantly decreased at 18 dpi (*p* < 0.01) and 21 dpi (*p* < 0.001) compared to those of the control group (Figure 1B). The thymus consists of a large number of thymocytes with four basic subpopulations. Thus, we measured variations in these thymocyte subpopulations after infection. The flow cytometric results showed the most dramatic decrease for CD4^+^CD8^+^ DP thymocytes; while this subset normally constitutes 80-85% of all thymocytes in control mice, these cells were almost absent at 21 dpi (Figure 1C). The DP thymocytes were not only reduced in proportion (Figures 1C and 1E right panel) but also in absolute numbers (Figures 1D and 1E left panel) at 18 dpi and 21 dpi. The results further showed that other subsets of thymocytes, such as CD4^−^CD8^−^ double negative (DN) and CD4^+^ single positive (SP) cells, were depleted. This decrease in thymocyte numbers paralleled the dramatic decline in thymic mass. Compared to TECs, thymocytes are the population undergoing the most apoptosis in the thymus. We used TUNEL assays to evaluate thymocytes apoptosis and found a significant increase in the number of TUNEL-positive cells in the thymus in the 18 dpi and 21 dpi groups (Figure 1F). The percentage of apoptotic cells was also calculated based on Annexin-V and PI staining and flow cytometry analysis, and the results showed that greater than 15% of the cells were labeled at 14 dpi, while only 8% labeled cells were observed in the control (Figure 1G). These results suggest that *A.C*. infection induces not only severe brain damage but also severe thymic atrophy accompanied by dramatic depletion of DP thymocytes through increased apoptosis. Thus, we next investigated whether these changes in thymocytes after *A.C*. infection were responsible for the observed thymic atrophy. Because thymocytes are supported by the TEC-constructed thymic microenvironment, we hypothesized that increased thymocyte death may be related to changes in thymic stromal cells (TECs). Therefore, we next evaluated the status of TECs after *A.C*. infection.

### *A.C.* infection reduced TECs through increased apoptosis

**Figure 2:**
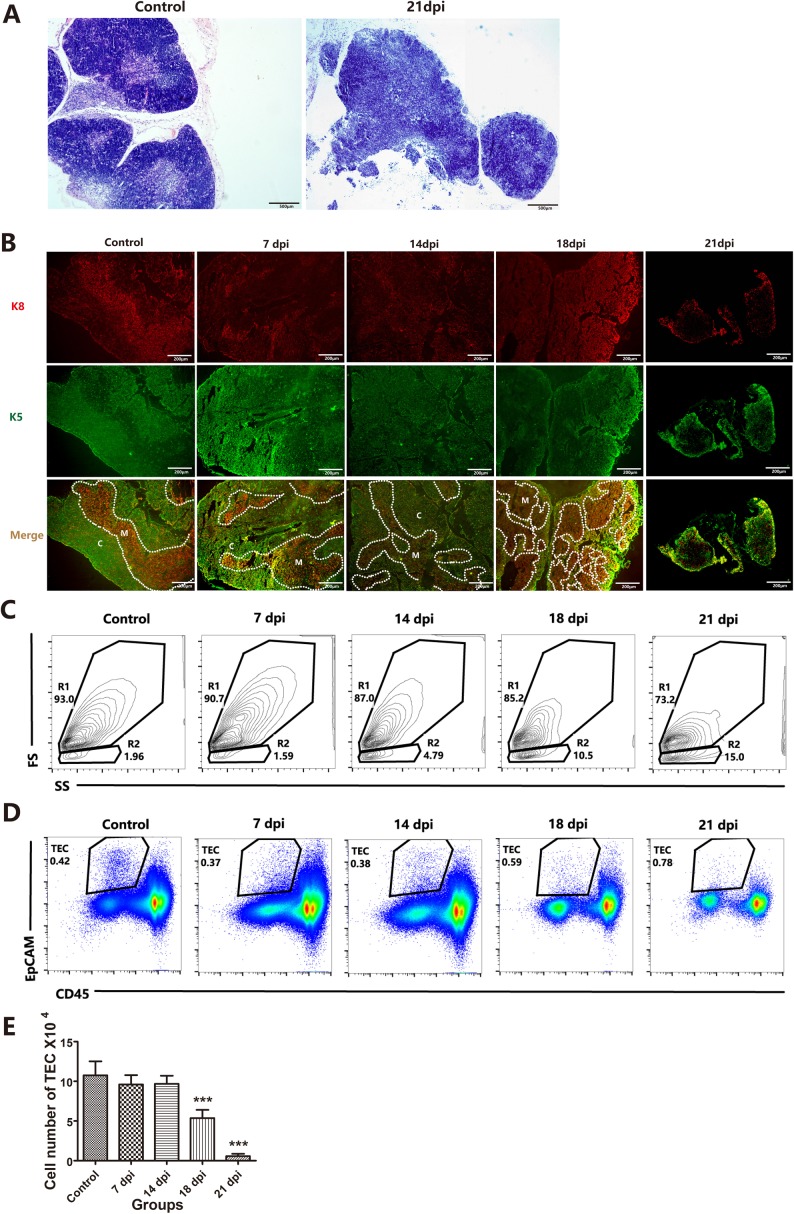
*A.C*. infection induced alterations of the thymic microstructure, accompanied by a loss of TECs **A**. H&E staining showing thymus histology of control and *A.C*.-infected (21 dpi) mice (original magnification, x5). **B**. Immunofluorescence staining of thymuses from mice stained for K5 and K8 (K5, green; K8, red). Broken lines indicate the CMJ (original magnification, x10). **C**. Representative flow cytometric dot plots (FS vs. SS) showing gating on live (R1) and dead and necrotic cells (R2). **D.** In the gated live cells, a TEC population within a gate of CD45-negative and EpCAM-positive events is shown. **E**. Cell numbers of TECs per mouse thymus are summarized in the graph. Data are representative of at least three independent experiments. The data are presented as the mean±S.D. ***p < 0.001.

To determine whether *A.C*. infection causes pathological changes in TECs, we used hematoxylin and eosin (H&E) staining to assess the morphology of the thymus. We found that the microstructure of the thymus at 21 dpi was clearly disrupted, displaying a dim cortico-medullary junction (CMJ) (Figure 2A, right panel) with a significantly reduced mass (Figure 2B rightmost panel). The atrophic phenotype gradually worsened with extended infection and was observed in both the thymic cortex and the thymic medulla. These results were confirmed by immunofluorescence staining of keratin (K) 5, a marker of the medulla, and K8, which indicates the cortical epithelial region (Figure 2B) [20]. A significant reduction in the entire thymic mass, associated with decreased total TECs (Figures 2C-2E), was observed in the thymus starting from 18 dpi (Figure 2E) compared with that of the control mice without infection. These results strongly indicated that *A.C.* infection induced TEC loss. TECs, as a 3D frame for the thymus, play important roles in supporting thymocyte development, thereby controlling the total thymic mass.

According to their localization, function, and molecular expression, TECs can be divided into two subsets, cortical TECs (cTECs) and medullary TECs (mTECs). cTECs constitute the cortex, where T cell precursors develop from the DN stages to the DP stage; mTECs constitute the medullary region, where thymocytes predominantly undergo negative selection [15, 21, 22]. We further evaluated whether *A.C*. infection influences specific TEC subsets.

**Figure 3:**
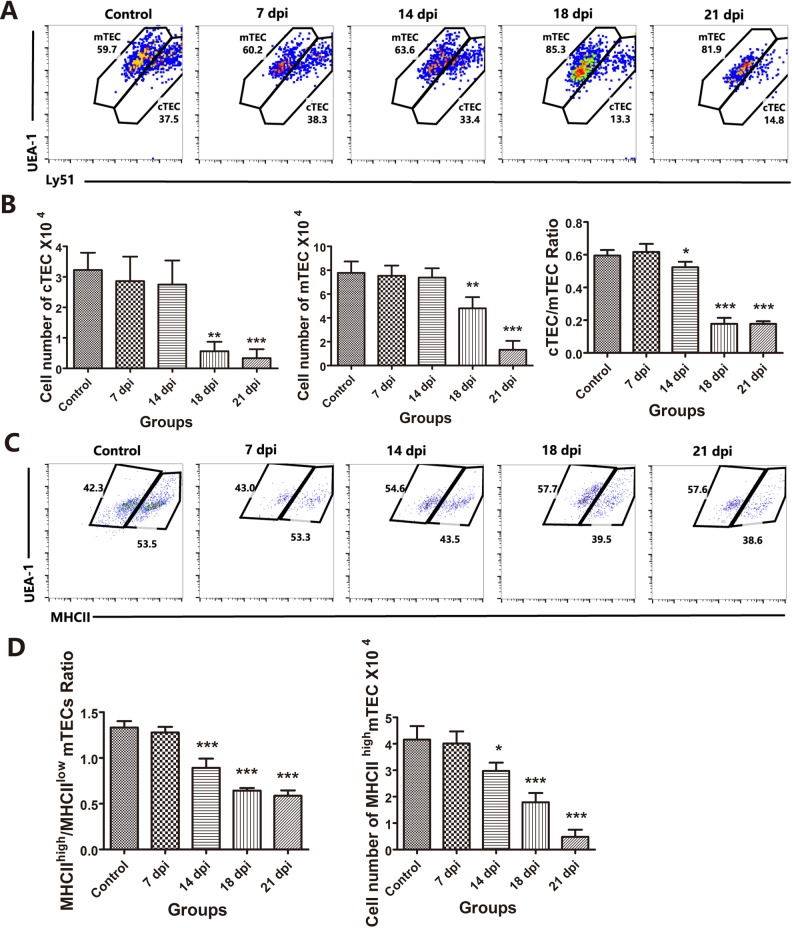
Changes in TEC subpopulations affected by *A.C*. infection **A**. Within the TEC gate, cTEC and mTEC populations are shown based on the level of Ly51 and UEA-1 expression. **B**. The cell numbers of cTECs and mTECs per mouse thymus, as well as the ratios of cTECs vs. mTECs, are summarized in the graph. **C**. A representative flow cytometric analysis shows the ratio of MHC-IIhigh vs. MHC-IIlow mTECs. **D**. A summary of the number of MHC-IIhigh mTECs and the ratio of MHC-IIhigh vs. MHC-IIlow mTECs. Data are representative of at least three independent experiments. The data are presented as the mean±S.D (*n* = 5). *p < 0.05, **p < 0.01, ***p < 0.001.

Although TECs share common characteristics (CD45^−^MHC-II^+^ and EpCAM^+^), these cells can be further divided based on the expression of Ly51 and UEA-1. cTECs show high expression of Ly51 and low expression of UEA-1, while mTECs show low expression of Ly51 and high levels of UEA-1. [23]. As shown in Figures 2D and 2E, CD45-negative and EpCAM-positive TEC numbers were dramatically decreased at 18 dpi and 21 dpi compared with those of the control group, and we then analyzed the changes in TEC subpopulations caused by *A.C*. infection. We found that both cTECs and mTECs at 18 dpi and 21 dpi, especially cTECs, were substantially decreased (Figure 3A) compared with those in the control group (Figure 3B, left and middle panels). The reduction of cTECs was also demonstrated by changes in the cTEC/mTEC ratio at 14 dpi (*p* < 0.05), with a slight reduction, and at 18 dpi and 21 dpi (*p* < 0.001), with a significant reduction, compared with that of the control group (Figures 3A and 3B, right panel). Figure 3A and 3B show that mTECs were less impaired than cTECs during *A.C.* infection.

mTECs can be divided into two subpopulations: MHC-II^high^ mature mTECs and MHC-II^low^ immature mTECs [24]. Because mTECs were less affected than cTECs, we further examined whether these two mTEC subpopulations were similarly affected by *A.C*. infection. The results showed that MHC-II^high^ mature mTECs were impaired with increasing duration of *A.C*. infection, showing a decreased ratio of MHC-II^high^ *vs*. MHC-II^low^ mTECs (Figures 3C and 3D). These findings suggest that mature mTECs might be more sensitive to *A.C*. infection-induced impairment.

**Figure 4:**
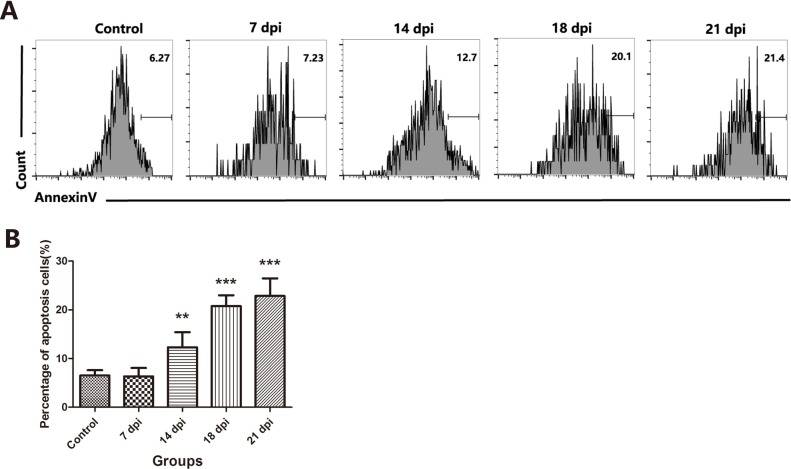
*A.C*. infection induced the loss of TECs due to increased apoptosis. A A representative flow cytometric analysis of Annexin V-positive cells in CD45-EpCAM+ TEC populations. **B**. A summary of the percentages of apoptotic cells in TECs. Data are representative of at least three independent experiments. The data are presented as the mean±S.D. **p < 0.01, ***p < 0.001.

Then, we sought to identify the changes that mediated the loss in TECs and whether this effect was caused by increased apoptosis. Based on the results of Annexin-V staining, we found that the number of Annexin-V-positive apoptotic TECs was increased 2-fold at 14 dpi and more than 3-fold at 18 dpi and 21 dpi (Figure 4). These results confirmed that *A.C.* infection induced the loss of both cTECs and mature mTECs through increased apoptosis, which may result in the observed thymic atrophy after *A.C.* infection.

### sAgs directly induced apoptosis of fetal thymic stromal cells and thymocytes

**Figure 5:**
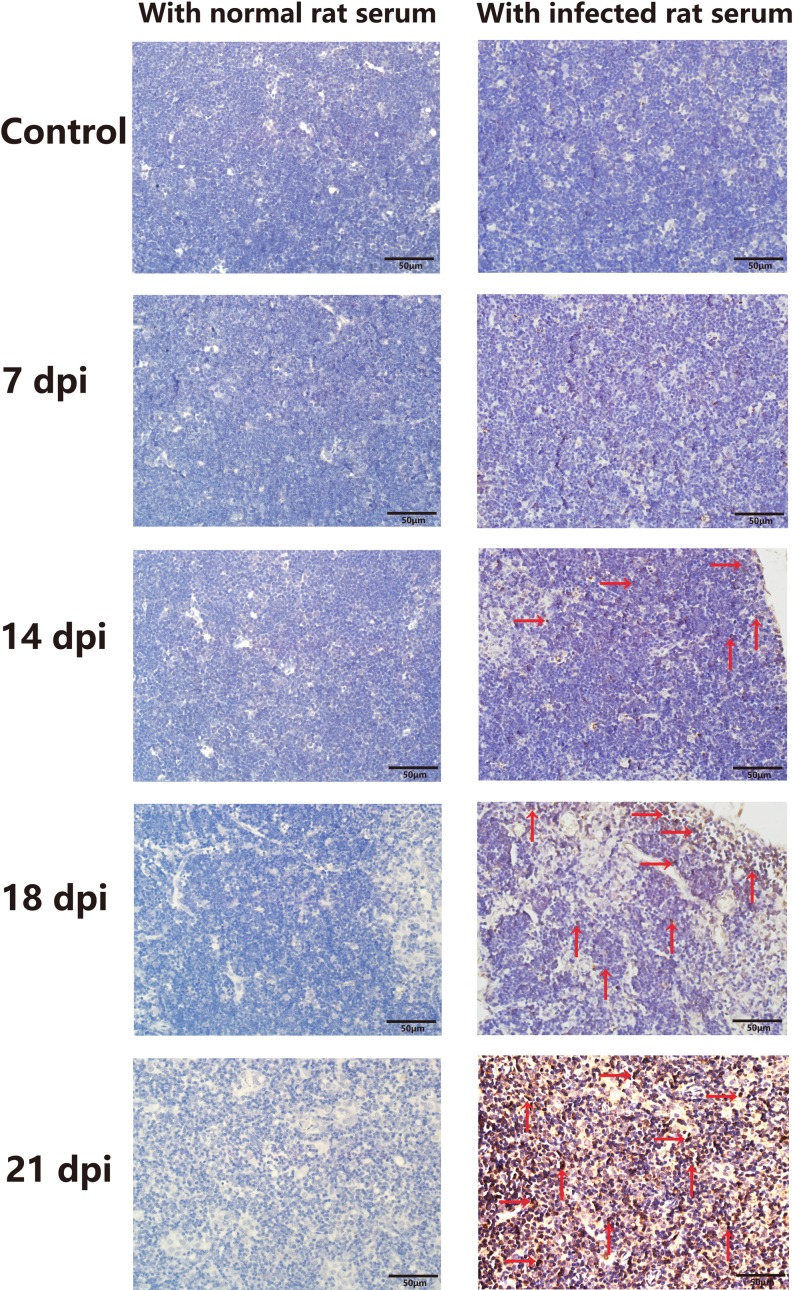
*A.C*. antigens were detected in the thymuses of infected mice *A.C*. antigen was detected in infected mouse tissues using rat serum containing anti-*A.C*. antibodies from an *A.C.-*immunized rat. *A.C*. antigen was present in the thymuses of infected mice (original magnification, x40) (*n* = 3). Red arrows show positive expression.

We next investigated the factors underlying the increased apoptosis of TECs during *A.C.* infection. *A.C*. worms do not directly invade the thymus to impair TECs during *A.C*. infection. However, during development and migration, the worms secrete sAgs into the host's circulatory system. In addition, disintegration of dead worms releases a large number of sAgs [25, 26]. Whether these sAgs of *A.C*. play a toxic role by entering the thymus and directly inducing TEC apoptosis and impairment remains unknown. To determine whether *A.C*. sAgs can be detected in the infected mouse thymus, we stained for sAgs using immunohistochemistry with *A.C*. protein*-*immunized rat serum, which contains anti-*A.C*. antibodies. Serum from normal rats was employed as a negative control. In slides of thymic tissues from infected mice (especially at 14 dpi, 18 dpi, and 21 dpi) incubated with *A.C.-*immunized rat serum, specific spots of brown staining, corresponding to *A.C*. sAg, were detected in infected thymic tissues but not in tissues incubated with normal rat serum (Figure 5). These findings indicated that *A.C.* antigen is present in the thymuses of infected mice.

**Figure 6:**
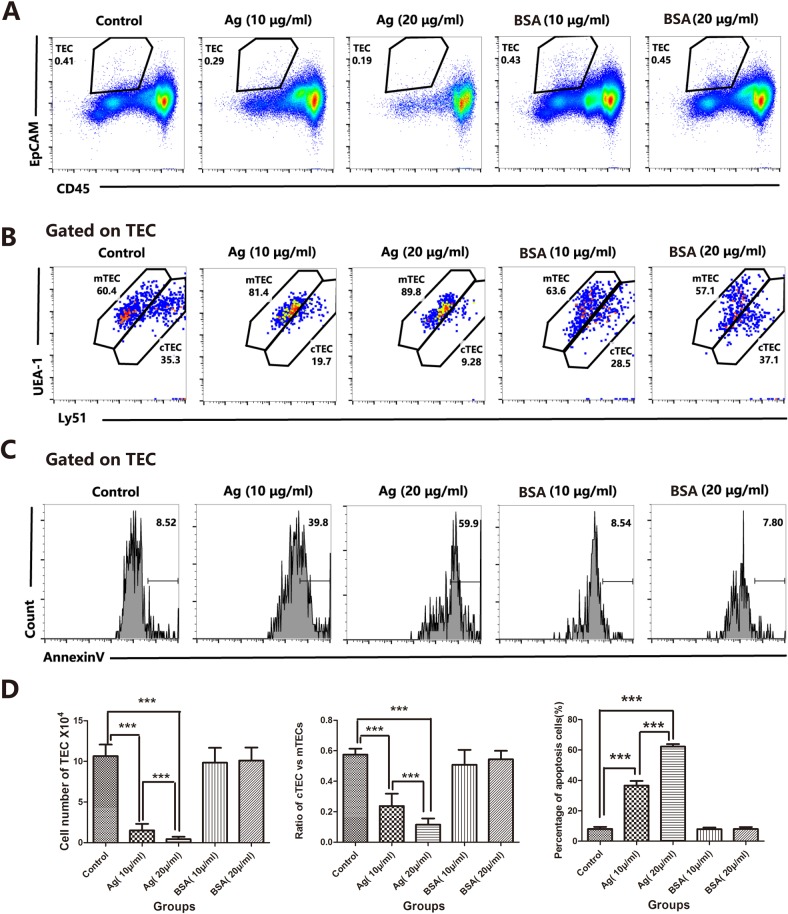
*A.C*. sAg stimulated the apoptosis of fetal thymic stromal cells in a fetal thymic organ culture **A**. Representative flow cytometric dot plots showing a gate of TECs (CD45-negative and EpCAM-positive events). **B**. Within the TEC gate, the mTEC and cTEC populations are separated based on the levels of Ly51 and UEA-1 expression (cTECs have higher expression of Ly51; mTECs have higher expression of UEA-1). **C**. A representative flow cytometric analysis of Annexin-V-positive cells in CD45-EpCAM+ TEC populations. **D**. A summary of the TEC cell numbers, the ratios of cTECs vs. mTECs and the percentages of apoptotic cells in TECs. Data are representative of at least three independent experiments. The data are presented as the mean±S.D. ***p < 0.001.

**Figure 7:**
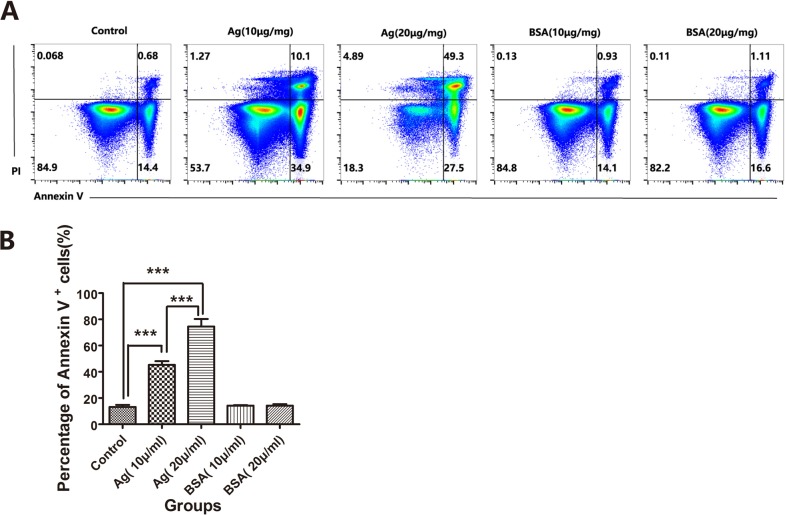
*A.C*. sAg stimulated thymocyte apoptosis in thymic mononuclear cell culture **A**. Thymic mononuclear cells isolated from normal mice were stimulated with soluble worm antigen (10 μg/ml or 20 μg/ml) for 6 h *in vitro*. The apoptotic thymocytes were identified by Annexin-V and PI staining. The FACS plots were gated on thymocytes. One representative FACS plot is presented, and the percentages indicate the proportions of apoptotic cells. **B**. A summary of the percentages of apoptotic cells in thymocytes, which is representative of three independent experiments, is shown. The data are presented as the mean±S.D (*n* = 5). ***p < 0.001.

To address whether these sAgs can directly impair thymocytes and TECs, we generated a fetal murine thymus organ culture (FTOC) system [27–29] and artificially added sAgs. FTOC is a useful culture system to observe the differentiation and development of thymocytes and TECs *in vitro* [29]. As expected, in the FTOC system, the sAgs directly increased the death and apoptosis of TECs, accompanied by decreased numbers of TECs and a reduced cTEC/mTEC ratio (Figures 6A-6D). These artificial sAg-induced changes were similar to the changes observed in the adult thymus following natural *A.C*. infection (Figures 2 and 3). In addition, we tested the effect of sAg on the isolated thymic stromal cells in culture. These isolated stromal cells were stimulated with sAgs (10 μg/ml or 20 μg/ml) for 6 h and then subjected to Annexin-V assays. The percentage of Annexin-V-positive cells was increased in a dose-dependent manner after sAgs stimulation compared with that in the control group (Figures 7A-7B).

**Figure 8:**
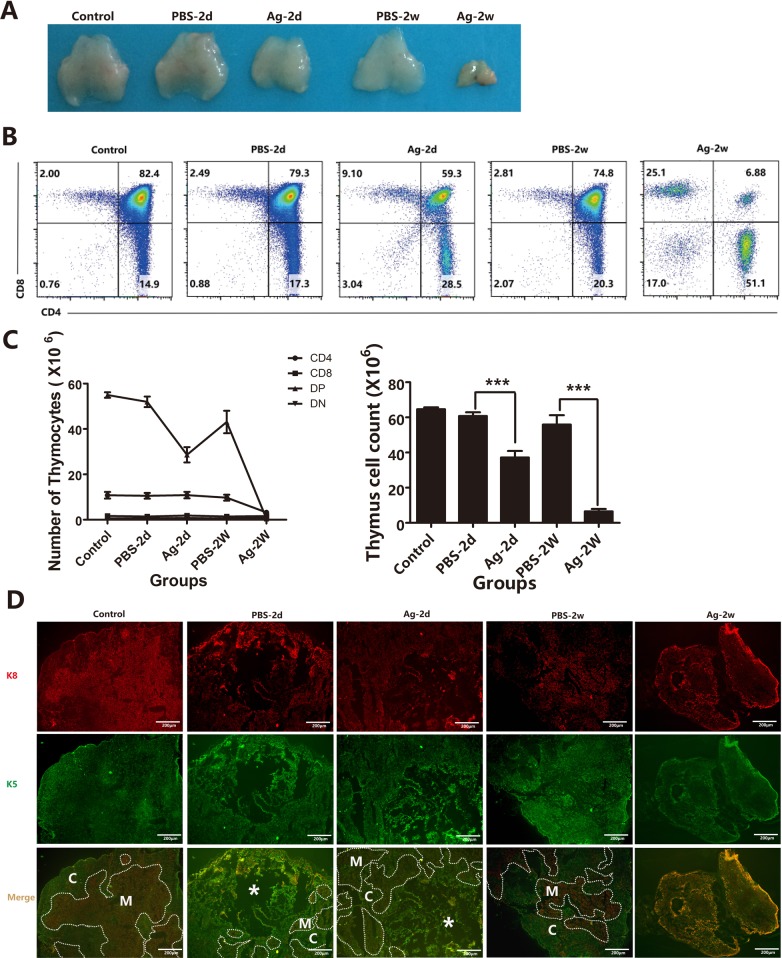
Intrathymic injection with soluble worm antigens from *A.C* induced thymic atrophy. Mice were divided into the following 5 groups: 1) Control: normal mice; 2) Group PBS-2d: mice with intrathymic injection of PBS (10 μl/mouse) were sacrificed 2 days after the injection; 3) Group Ag-2d: mice with intrathymic injection of soluble worm antigen (1 μg in 10 μl/mouse) were sacrificed 2 days after the injection; 4) Group PBS-2w: mice with intrathymic injection of PBS (10 μl/mouse) were sacrificed 2 weeks after the injection; and 5) Group Ag-2w: mice with intrathymic injection of soluble worm antigen (1 μg/10 μl) were sacrificed 2 weeks after the injection. **A**. A representative image shows the mass of the thymus. **B**. Changes in the ratios of different thymocyte subsets (DN, SP and DP) are shown, as analyzed by flow cytometry, from different groups. One representative FACS plot is presented, and the percentages indicate the proportions of DN, CD8+SP, CD4+ SP and DP cells. **C**. Changes in the numbers of total thymocytes and DN, SP and DP thymocytes from different groups. **D**. Immunofluorescence analysis of thymus tissues stained for K5 and K8 (K5, green; K8, red). Broken lines indicate the CMJ (original magnification, x10). Data are representative of at least three independent experiments. The data are presented as the mean±S.D. ***p < 0.001. White asterisk represents injection position for the immunofluorescence results.

**Figure 9:**
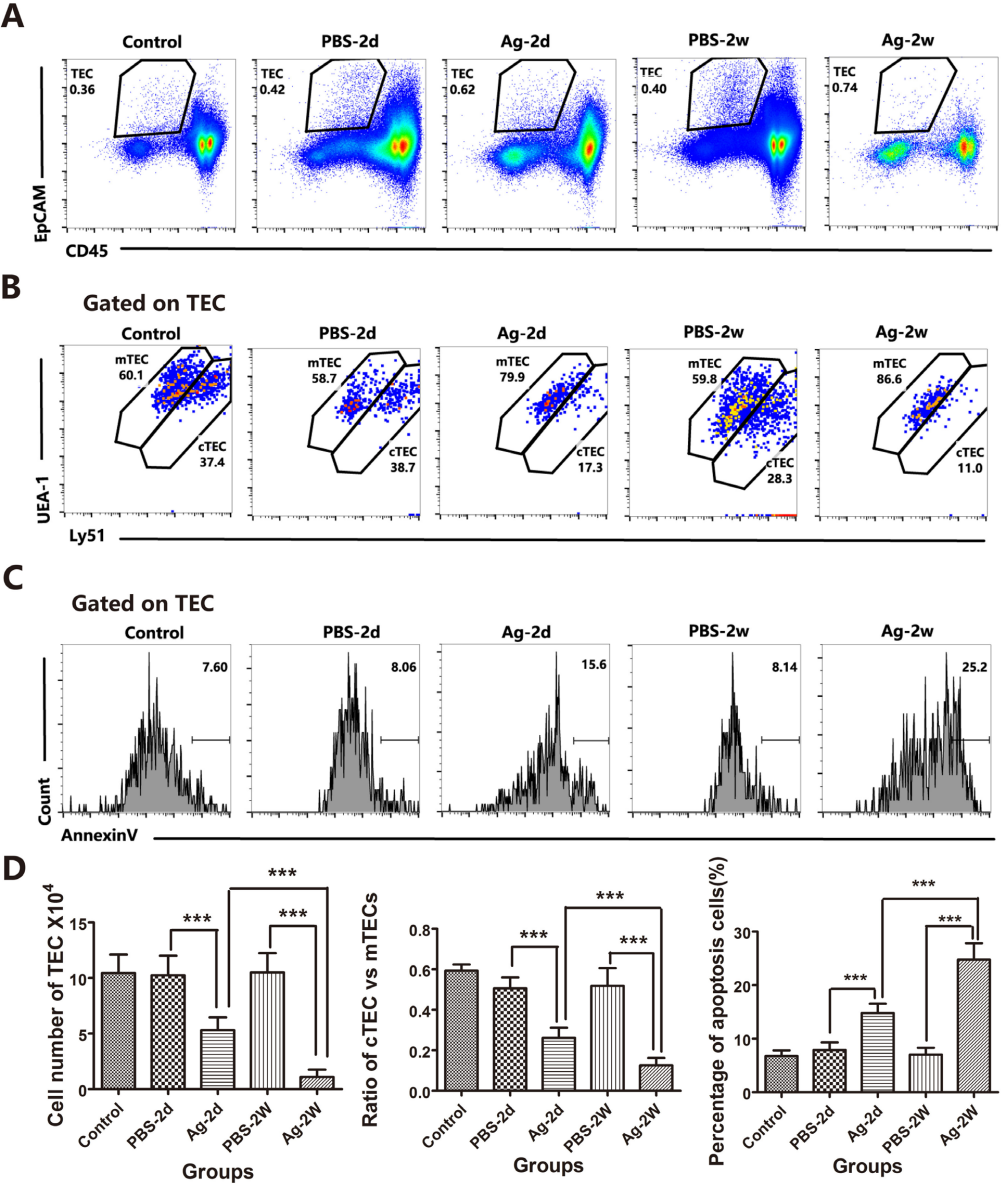
Soluble worm antigens from *A.C*. induced increased apoptosis in TECs from intrathymic-injected mice Mice were divided into groups as described above. **A**. TEC cells are shown in a gate of CD45-negative and EpCAM-positive events. **B**. Within the TEC gate, cTEC and mTEC populations are shown based on the levels of Ly51 and UEA-1 expression. cTECs have higher expression of Ly51; mTECs have higher levels of UEA-1. **C**. A representative flow cytometric analysis shows Annexin-V-positive cells in the CD45-EpCAM+ TEC populations. **D**. A summary of the numbers of TECs, the ratios of cTECs vs. mTECs and the percentages of apoptotic cells in the TEC population. Data are representative of at least three independent experiments. The data are presented as the mean±S.D. ***p < 0.001.

In addition to demonstrating the direct effect of sAgs on TECs *in vitro*, we also performed *in vivo* experiments using intrathymic injection of sAgs, rather than live *A.C*. infection, in adult mice. The results showed that intrathymic injection of *A.C.* sAgs directly induced severe thymic atrophy, especially 2 weeks after injection (Figure 8A). Flow cytometry analysis showed that the ratio of DP thymocytes was also dramatically decreased at 2 days after injection, and the DP thymocytes were nearly absent (< 10%) at 2 weeks after injection. Furthermore, the numbers of DP thymocytes were decreased (*p* < 0.001) at 2 days after injection, and the numbers of CD4^+^ SP (*p* < 0.005) and DN thymocytes (*p* < 0.001) showed significant depletion at 2 weeks after injection (Figures 8B and 8C, left panel). Additionally, the total number of thymocytes was dramatically decreased, leading to a 33.3% and 91.3% reduction at 2 days and 2 weeks after injection, respectively (Figure 8C, right panel). Immunofluorescence assays showed a significant reduction in the total thymic mass, with reduced thickness in both of the cortical and medullary layers at 2 weeks after injection, compared with that of control mice (intrathymic injection with PBS) (Figure 8D). Intrathymic injection with *A.C*. sAgs also severely impaired the TEC subpopulations (Figure 9), and we found a reduction in the absolute numbers of both cTECs and mTECs and a decreased mTEC/cTEC ratio compared with those of the control group (Figures 9A-9B). Furthermore, there was an increase in apoptotic TECs (Figure 9C) following the injection of sAgs, which was similar to the natural infection-induced changes (Figures 2 and 3). Together, these data conclusively demonstrate that sAgs from *A.C*. directly induce local impairment and thymic atrophy by promoting apoptosis of TECs and thymocytes, even without live *A.C*. infection.

## DISCUSSION

In this study, we found that *A.C.* larvae-induced brain impairment in non-permissive hosts caused severe thymic atrophy, characterized by increased thymocyte apoptosis and altered T cell subpopulations, particularly the DP subset. Additionally, infection severely impaired TECs, especially cTECs and mature mTECs, the thymocyte microenvironment, and thymocyte survival and maturation. In addition to the previously reported indirect promotion of thymocyte death by the HPA axis, our study showed that decreased numbers of thymocytes after *A.C*. infection may not be the reason for, but instead the outcome of, defects in TECs due to a direct impairment by *A.C*. sAgs in the thymus of infected mice. These sAgs also directly depleted thymocytes through a non-HPA axis during *A.C*. infection. Notably, TEC damage-induced thymic atrophy was irreversible.

The thymus is the central immune organ of thymocyte maturation and selection, resulting in the generation of naive T cells, which migrate to peripheral lymphoid tissues and play important roles in host immune defense [30]. However, the thymus is a common target organ in infectious diseases [31], and several pathogens are known to cause thymic atrophy. Pathogen infection-induced acute thymic atrophy affects systemic cellular immune function, resulting in delayed clearance of pathogens, which is particularly harmful [32]. It is essential to determine the specific pathways in distinct situations to prevent thymic atrophy and infection-induced immune deficiency [33]. However, the mechanisms involved in infection-induced thymic atrophy remain poorly defined.

Some viruses, bacteria, and parasitic protozoa directly invade the cells or tissues of the thymus to destroy the normal structure and/or function during infection, while also changing the levels of soluble factors in the circulatory system of the host or the antigens present within the thymus to alter T cell differentiation [12]. This systemic and/or local effect plays an important role in infection-induced thymic atrophy [9]. Systemic effects occur when soluble factors, such as GCs and other pro-inflammatory mediators, are released into the bloodstream [7, 12]. GCs have been reported to play major roles in *Trypanosoma cruzi*-induced thymocyte death [10]. Thymocytes, especially DP thymocytes, show high sensitivity with a strong apoptotic response to GCs due to GR expression on the thymocyte surface [34, 35]. Additionally, GCs play alternative roles in inducing thymocyte survival by modifying T cell antigen receptor-induced signals [36, 37]. Our collaborator previously reported that *A.C*. infection in the brain increases the expression of GCs in serum and that infection-induced progressive thymic atrophy was paralleled by increased circulating levels of GCs. These authors also proposed that HPA-axis activation contributes to immunosuppression and might be connected with the decrease in lymphocytes observed during thymic atrophy after *A.C*. infection [6]. However, blockade of GRs could not stop the thymocyte decrease during *A.C*. infection [6], and thus, the precise mechanisms for *A.C*.-induced thymic atrophy cannot be explained. Additionally, the high level of pro-inflammatory cytokines was suggested to play an important role in infection-induced thymic atrophy. Important systemic pro-inflammatory cytokines, including interleukin-1 (IL-1), tumor necrosis factor (TNF), and IL-6, activate nuclear factor-kappa B (NF-κB), a crucial signaling molecule involved in the immune response and thymocyte development [38, 39]. GCs are steroidal anti-inflammatory agents with anti-inflammatory effects [18]; however, it remains difficult to explain why *A.C*. infection-induced high GC expression is associated with severe inflammation.

The thymic matrix is composed of TECs, which are significant components of the thymic microenvironment and can support the development of all stages of thymocytes [31]. Thymocytes contact TECs to form a complex network of stromal cell support during development and maturation [40, 41]. The development of DP cells predominantly occurs in the cortex, while we found that cortical TECs were the major subset impaired during *A.C*. infection. Therefore, we hypothesized that the dramatic and selective loss of DP thymocytes was due to deterioration of the cortical thymic microenvironment and not to the increased HPA-axis activation. Because DP cells do not have more GC receptor than other subpopulations, they are less likely to be affected by selective loss through HPA-axis activation.

Due to the lack of GR expression, GCs cannot affect TECs directly [16]. Therefore, TEC defects during the infection must be induced by other factors. Several viruses directly infect TECs and alter their function by inducing cell apoptosis/death, thereby influencing thymocyte differentiation [42]. Other reports have shown that specific microbial factors, such as lipopolysaccharide (LPS), can directly promote thymocyte/TEC death [43, 44]. In a non-permissive host, the *A.C*. stage-*III* larvae can develop to stage-*IV* larvae. As a nematode and extracellular pathogen, all stage-*III* and stage-*IV* larvae fail to invade the thymuses of non-permissive hosts. However, the larvae continually excrete sAgs during development and migration. Meanwhile, the host's immune system attacks the larvae, and the dead larvae also release a large amount of sAgs. We reported that soluble antigens derived from stage-*IV A.C*. larvae contain various proteins that play diverse functions during infection [19, 26, 45]. In our study, sAgs from *A.C*. were detected in the thymus after infection, which may be due to the circulation of sAgs from larvae in the brain. Furthermore, we found that sAgs had a local/direct effect on TECs and confirmed that the loss of TECs was due to sAgs inducing TEC apoptosis, based on experiments using FTOC co-culture with *A.C.* sAgs. The deterioration of TECs disrupts the thymic epithelial network and alters the thymic microenvironment, subsequently inducing apoptosis of thymocytes. Furthermore, we found that sAgs directly promoted thymocyte apoptosis, which is another direct cause of thymic atrophy, based on experiments using thymic mononuclear cell cultures stimulated with sAgs. Thymic lobes injected with sAgs *in vivo* showed severe thymic atrophy, accompanied by a significant decrease in thymocytes and increase in apoptotic TECs, similar to *A.C.* infection-induced thymic atrophy. Therefore, these sAgs (from *A.C*.) showed direct local effects on thymic atrophy that were not mediated by the HPA axis. However, our current understanding of the complex mechanism underlying thymic atrophy during *A.C*. infection is limited, and cross-talk between different pathways may occur. Further studies will focus on the specific soluble compounds or molecules constituting the sAg pool of *A.C*., which should help to clarify this complex mechanism.

In general, our study revealed a new mechanism by which *A.C.* infection induces thymic atrophy in non-permissive hosts. *A.C*.-derived sAgs either impair TECs to disrupt the thymic microenvironment, and subsequently affect thymocytes survival and maturation, or directly impair thymocytes through a non-HPA axis. Notably, the mechanism related to both these impairments is not mediated through the HPA axis. Our results further emphasize that parasitic infection not only causes local injury but also has an enormous impact on the immune system via the dissemination of antigens through the circulatory system. Our work may help elucidate other diseases with thymic atrophy in young patients and provide a rationale for greater clinical attention on immune system recovery during angiostrongyliasis and other nervous system diseases.

## MATERIALS AND METHODS

### Ethics statement

All research involving mice and parasites in our study was conducted in accordance with the relevant regulations and guidelines of the Ethics Committee of Sun Yat-sen University, and all animal care protocols were approved by the Ethics Committee of Sun Yat-sen University.

### Mice, infections and antigen preparation

BALB/c mice (8 weeks old, male) were purchased from the Laboratory Animal Center of Sun Yat-sen University (Guangzhou, China). Each mouse was orally infected with 30 third-stage larvae. The stage-*III* larvae of *A.C*. were collected from the tissues of infected *Biomphalaria straminea* using the method previously described by our lab [46]. The mice were euthanized on days 7, 14, 18, and 21 after infection with *A.C*., and we defined these time-point in terms of dpi. Each group contained at least 5 mice, and three independent experiments were performed. *A.C*. sAgs were extracted from purified stage-*IV* larvae derived from the brains of mice at 21 dpi and were prepared as previously described [26]. After grinding, freeze/thaw, filter sterilization, removal of endotoxins and protein concentration determination, the sAg was stored at −80°C.

### Histological examination, immunofluorescence and immunohistochemistry analysis

The brain and thymus tissues were fixed, cut into 5-μm-thick sections and stained with H&E. For immunofluorescence analysis, cryosections of thymus tissue (6-μm-thick) were fixed in cold acetone and blocked with 3% bovine serum albumin in Tris-buffered saline (TBS). The primary antibodies used were specific for cytokeratins 5 and 8. The secondary antibodies used were Alexa Fluor 488 AffiniPure donkey anti-rat IgG (H+L) and Alexa Fluor 594-conjugated AffiniPure goat anti-Armenian hamster IgG (H+L) (Jackson ImmunoResearch Lab). Thymic apoptotic cells were detected by TUNEL staining using the In Situ Cell Death Detection Kit, POD (Roche Applied Science, Mannheim, Germany) according to the manufacturer's guidelines. For immunohistochemistry, 5-μm-thick thymus sections were incubated in *A.C.-*infected rat serum (1: 200 dilution), which contained the anti-*A.C*. IgG antibody, in a humidified chamber at 4°C overnight. Serum from normal rats was employed as a control. Then, these sections were probed with HRP-conjugated rabbit anti-rat IgG (1:800 dilution, Proteintech., USA) at RT for 1 h. The DAB reagent was used to develop immunoreactive signals. Finally, after the sections were counterstained with Mayer's hematoxylin, they were dehydrated, cleared in xylene and mounted. For each group, at least three samples were evaluated. All slices were examined using microscopy (Olympus BX63).

### Cell preparation, antibodies and flow cytometry analysis

Thymocytes filtered through a cell strainer (200 μm) were stained with PE-Cyanine5-conjugated CD4 or FITC-conjugated CD8 antibody (eBioscience). For the apoptosis detection assay, the cells were stained with Annexin-V and propidium iodide (PI), according to the manufacturer's instructions (BD Biosciences). Enzymatically (Collagenase-V/DNase-I) digested thymic cells, enriched for TECs [47], were stained with PE-Cyanine7-conjugated CD45, APC-conjugated EpCAM, eFluor 450-conjugated MHC class II, and PE-conjugated Ly51 antibodies (all from eBioscience), as well as UEA-1 lectin labeled with FITC (Vector Laboratories). Data were acquired on a FACScan flow cytometer or Gallios (all from Beckman Coulter) and analyzed using FlowJo software (Treestar, San Carlos, CA).

### The FTOC system

The FTOC system was prepared as described previously [48]. Briefly, thymic lobes dissected from 1- to 2-day-old newborn mice (BALB/c) were cultured for 2 days on top of Nucleopore filters (Whatman) placed in RPMI-1640 medium supplemented with 2% fetal bovine serum (FBS) (Life Technologies). For analysis of the direct effect of soluble antigens on TECs, sAg (10 μg/ml or 20 μg/ml) was added to the culture medium. PBS was used to establish the control group, and bovine serum albumin (BSA) (10 μg/ml or 20 μg/ml) was used to establish the negative protein control group.

### Thymus mononuclear cell culture system

Filtered thymocytes (total 1×10^6^ cells/well, 24-well cell culture plates) were stimulated with sAgs (10 μg/ml or 20 μg/ml) in 10% (v/v) RPMI-1640 medium containing FBS and supplemented with 50 U/ml penicillin and streptomycin and incubated for 6 h. PBS was used to establish the control group, and BSA (10 μg/ml or 20 μg/ml) was used to establish the negative protein control group.

### Intrathymic injection

After receiving anesthesia, mice were placed in the supine position, and a 0.5-cm small incision was made on the skin and manubrium to expose the thymus as described previously [49]. Then, 10 μl (1 μg) of sAg from *A.C*. in suspension was injected into each thymic lobe using a Hamilton syringe (Fisher Scientific). For the sham-operated mice, 10 μl PBS was injected into each thymic lobe. The surgery survival rate was >99%.

### Statistical analysis

Data are presented as the mean±standard deviation (S.D.). Differences between groups were analyzed with the independent samples t test or one-way analysis of variance (ANOVA) using SPSS 16.0. The data were analyzed, and *p* < 0.05 was considered significant.

## SUPPLEMENTARY MATERIAL FIGURE



## Abbreviations

*A.C.*: *Angiostrongylus cantonensis*
CNS: central nervous system
HPA: hypothalamus-pituitary-adrenal
TEC: thymic stromal/epithelial cell
sAgs: soluble antigens
DP: double positive
GR: glucocorticoid receptor
GC: glucocorticoids
dpi: day post infection
cTECs: cortical thymic epithelial cells
mTECs: medullary thymic epithelial cells
FTOC: fetal murine thymus organ culture system
