# Supplementary material for: Soluble antigens from the neurotropic pathogen Angiostrongylus cantonensis directly induce thymus atrophy in a mouse model
Source: Oncotarget. 2017 May 12;8(30):48575–90. doi: 10.18632/oncotarget.17836 (PMC5564709; doi:10.18632/oncotarget.17836)
Supplement: Supplementary file 1 [file oncotarget-08-48575-s001.pdf]

Soluble antigens from the neurotropic pathogen *Angiostrongylus cantonensis* directly induce thymus atrophy in a mouse model

Supplementary Material

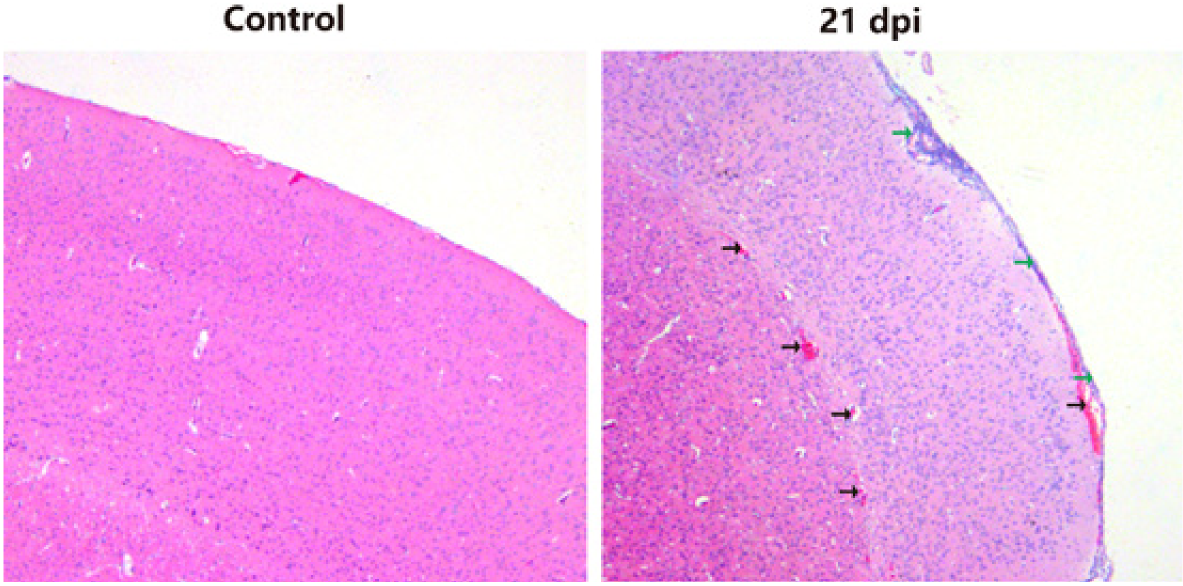

Supplementary Figure S1. Pathological status in the brains of mice with *A.C.* infection. Brain tissues were collected for pathology and evaluated by H&E (hematoxylin and eosin) staining. Images are shown using an automatic upright microscope (AxioImager Z1) at x20 magnification. Black arrows show hemorrhages, and green arrows show inflammatory cell infiltration. The results are representative of at least three independent experiments. Each group contained 5-6 mice.
